# Use of back-scatter electron signals to visualise cell/nanowires interactions *in vitro* and *in vivo*; frustrated phagocytosis of long fibres in macrophages and compartmentalisation in mesothelial cells in vivo

**DOI:** 10.1186/1743-8977-9-34

**Published:** 2012-08-28

**Authors:** Anja Schinwald, Ken Donaldson

**Affiliations:** 1 Centre for Inflammation Research, Queen’s Medical Research Institute, MRC/University of Edinburgh, 47 Little France Crescent, Edinburgh, EH16 4TJ, UK

**Keywords:** Backscatter scanning electron microscopy (BSE), Frustrated phagocytosis, THP-1 macrophages, Pleural macrophages, Parietal pleura mesothelium

## Abstract

**Background:**

Frustrated phagocytosis has been stated as an important factor in the initiation of an inflammatory response after fibre exposure. The length of fibrous structures has been linked to the potential of fibres to induce adverse health effects for at least 40 years. However, we only recently reported for the first time the threshold length for fibre-induced inflammation in the pleural space and we implicated frustrated phagocytosis in the pro-inflammatory effects of long fibres. This study extends the examination of the threshold value for frustrated phagocytosis using well-defined length classes of silver nanowires (AgNW) ranging from 3–28 μm and describes in detail the morphology of frustrated phagocytosis using a novel technique and also describes compartmentalisation of fibres in the pleural space.

**Methods:**

A novel technique, backscatter scanning electron microscopy (BSE) was used to study frustrated phagocytosis since it provides high-contrast detection of nanowires, allowing clear discrimination between the nanofibres and other cellular features. A human monocyte-derived macrophage cell line THP-1 was used to investigate cell-nanowire interaction *in vitro* and the parietal pleura, the site of fibre retention after inhalation exposure was chosen to visualise the cell- fibre interaction *in vivo* after direct pleural installation of AgNWs.

**Results:**

The length cut-off value for frustrated phagocytosis differs *in vitro* and *in vivo*. While *in vitro* frustrated phagocytosis could be observed with fibres ≥14 μm, *in vivo* studies showed incomplete uptake at a fibre length of ≥10 μm. Recently we showed that inflammation in the pleural space after intrapleural injection of the same nanofibre panel occurs at a length of ≥5 μm. This onset of inflammation does not correlate with the onset of frustrated phagocytosis as shown in this study, leading to the conclusion that intermediate length fibres fully enclosed within macrophages as well as frustrated phagocytosis are associated with a pro-inflammatory state in the pleural space. We further showed that fibres compartmentalise in the mesothelial cells at the parietal pleura as well as in inflammatory cells in the pleural space.

**Conclusion:**

BSE is a useful way to clearly distinguish between fibres that are, or are not, membrane-bounded. Using this method we were able to show differences in the threshold length at which frustrated phagocytosis occurred between *in vitro* and *in vivo models*. Visualising nanowires in the pleura demonstrated at least 2 compartments – in leukocyte aggregations and in the mesothelium - which may have consequences for long term pathology in the pleural space including mesothelioma.

## Background

The toxicology of fibres is a sub-specialty of particle toxicology developed in response to the asbestos experience [1] but whose tenets have been used to quantify the hazards from vitreous fibres [2] organic fibres such as p-aramid [3] and most recently nanofibres [4,5]. Current understanding of the fibre hazard is based on the ‘fibre pathogenicity paradigm’ which has predictive power and is the most robust structure/ toxicity relationship in particle toxicology. The paradigm identifies thinness, length and biopersistence as the 3 features that determine the pathogenic potential of any fibre sample [5]. Thinness determines respirability, that is the likelihood that a fibre penetrates the respiratory tract beyond the ciliated airways [3] where most damage is likely to occur, whilst biopersistence determines whether the fibre will retain its structure integrity i.e. fibrous shape, during residence in the lung. If the fibre is composed of soluble components that are leached from the fibre under the conditions it encounters *in vivo*, then the fibre is likely to become weakened, break and become part of the short fibre pool. Short fibres are not pathogenic in the fibre sense, although they may have harmful effects as particles, whilst long fibres do cause fibre-type pathogenicity; this length-dependent pathogenicity has been demonstrated in numerous studies *in vivo*[6-8] and *in vitro*[9-11].

**Figure 1:**
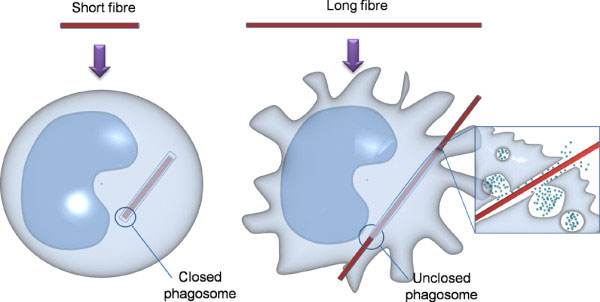
**Diagrammatic representation of short fibre complete phagocytosis and long fibre –mediated frustrated phagocytosis.** Short fibres can be fully phagocytosed by macrophages whereas long fibres are too long to be fully taken up leading to an unclosed membrane and leakage of cell content.

One major arbiter of length-dependent pathogenic effect is the alveolar macrophage and pleural macrophage whose normal function is to phagocytose fibres and clear them. Because of the unusual aerodynamics of fibres, extremely long fibres penetrate and deposit beyond the ciliated airways [3]. Macrophages that attempt to phagocytose long fibres cannot enclose them leading to ‘frustrated phagocytosis’ as shown diagrammatically in Figure 1; in contrast short fibres are fully enclosed in the phagosomes (Figure 1).

We recently reported length-dependent effects of a range of fibres including asbestos [8], carbon nanotubes [12,13], silver nanowires (AgNW) [14] and nickel nanowires (NiNW) [11] at the peritoneal and pleural mesothelial surfaces. All of these fibre types appear to comply with the fibre pathogenicity paradigm with length-dependent effects in the pleural space as a result of retention at stomata (3–10 μm in diameter) on the parietal pleura and frustrated phagocytosis. These data show persuasively that the length threshold for pleural retention of any fibres is 5 μm and that fibre shorter than this threshold are not retained and do not cause inflammation [14].

Whilst we evoked frustrated phagocytosis in the pro-inflammatory effects, we also noted that intermediate 5 μm length fibres, were fully enclosed by macrophages but were pro-inflammatory, as previously recorded [14]. Due to restrictions on space we could not fully explore the morphological basis of this new effect and here we extend these findings to fully characterise fibre length effects particularly frustrated phagocytosis *in vitro* and *in vivo*. We utilised the technique of backscatter electron microscopy in particular to investigate this phenomenon. We also describe the retention of fibres in the parietal mesothelial layer, an anatomical region not hitherto identified a retention compartment for fibres.

## Results

### AgNW panel

**Figure 2:**
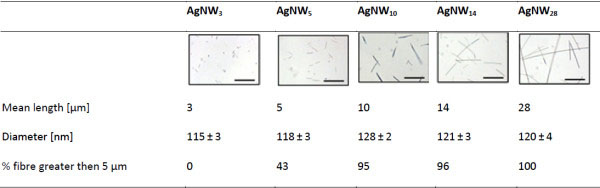
**Characteristics of AgNW panel.** Light microscopy images of AgNW panel in 0.5% BSA/saline. Scale bar 20 μm.

Characteristic of the AgNW panel are summarised in Figure 2. The images illustrate the uniform distribution of the AgNWs with no aggregation being present. Complete characterisation of the AgNW panel was described previously by Schinwald *et al*. [14].

### Membrane integrity and metabolic activity in the *in vitro* model

**Figure 3:**
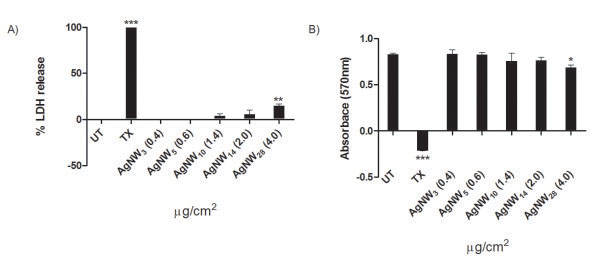
**THP-1 membrane integrity and proliferation/metabolic activity.A**) Measurement of membrane integrity plotted as % LDH release based and positive and negative control. Only AgNW_28_ lead to a significant release of LDH into the supernantant. **B**) Proliferation and metabolic activity was measured using alamarBlue^®^. Cell treated with AgNW_28_ showed decreased proliferation. Significance versus vehicle control *P <0.05, **P <0.01. Data represent mean ± s.e.m. of n = 5.

The *in vitro* experiments were based on a treatment dose which was non/low-toxicity to THP-1 cells in culture adjusted for each fibre length to produce approximately the same fibre number (Table 1). The 2 μg/cm^2^ dose based on AgNW_14_ was determined by measuring the membrane integrity via the release of lactate dehydrogenase (LDH) into the supernatant and cell proliferation of the THP-1 cells after 24 hour treatment; however, AgNW_28_ caused a significant decrease in membrane integrity and loss of proliferation and metabolic activity (Figure 3A,B).

**Table 1:** Calculation for the mass adjustments for equalisation of number

**Length class [μm]**	**Calculation to equalise for the same fibre number**	**Dose (μg/cm**^**2**^**)**
**3**	3/14 × 2	0.4
**5**	5/14 × 2	0.6
**10**	10/14 × 2	1.4
**14**	standard	2.0
**28**	28/14 × 2	4.0

### Bright field microscopy of THP-1 cells after 4 hour exposure *in vitro*

**Figure 4:**
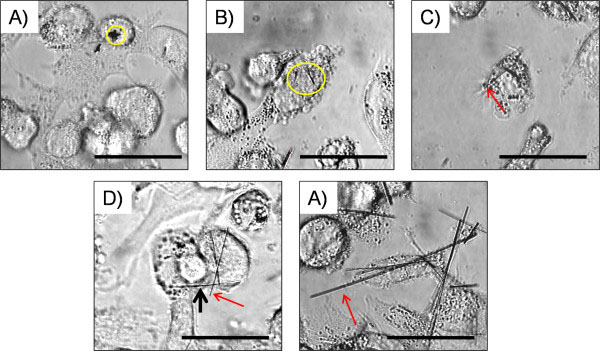
**Bright-field microscope image of THP-1 cells treated with AgNWs. A**) AgNW_3_ and B) AgNW_5_ were fully enclosed by the cell as indicated by the yellow circle. **C**) AgNW_10_ were mostly taken up, however a small number of fibres were not fully enclosed and fibre ends were protruding out of the cells as indicated by the red arrow. **D**) AgNW_14_ and **C**) AgNW_28_ caused frustrated phagocytosis. Red arrows indicate fibres partly taken up by THP-1 cells and the black arrow indicates sharing of a fibre between two adjacent cells. Scale bar = 20 μm.

**Figure 5:**
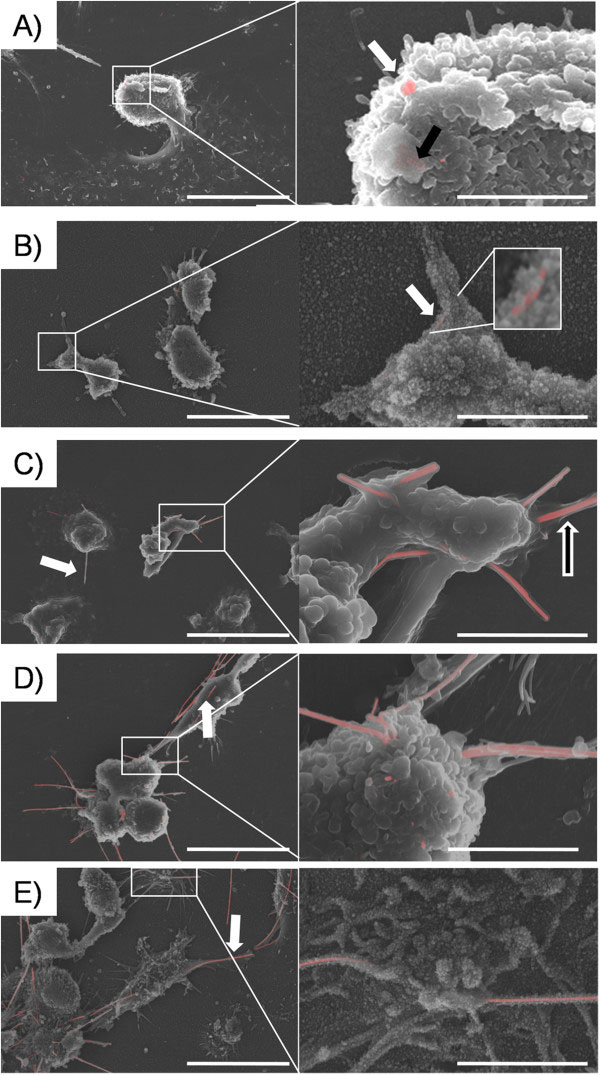
**BSE images of differentiated THP-1 cells treated for 4 hours with AgNW.A**) AgNW_3_ treated macrophages showed full phagocytosis of the nanowires. Only one nanowire could be visualised outside a cell (white arrow) and a few underneath the surface indicated by the black arrow. **B**) After AgNW_5_ treatment all nanowires were phagocytosed by cells after 4 hours (white arrow). **C**) A big proportion of AgNW_10_ appear membrane bound and therefore phagocytosed by cells (black/white arrow), however a few fibres could be seen protruding out of cells (white arrow). Frustrated phagocytosis was observed after AgNW_14_ (**D**) and AgNW_28_ (**E**) treatments, since most of fibres can be seen protruding out of the cells or shared between cells (white arrow and insert). BSE merged image with a scale bar of 10 μm and 5 μm for the inserts.

THP-1 cells were exposed to the panel of AgNWs for 4 hours and bright field images were taken using a 60× magnification. THP-1 cells completely phagocytose AgNW_3_ (Figure 4A) and AgNW_5_ (Figure 4B). The fibres were fully taken up as indicated by the yellow circle. AgNW_10_ were mostly phagocytosed with only a small percentage of fibre-ends protruding out of the cells as indicated by the red arrow (Figure 4C). Frustrated phagocytosis was observed after treatment with AgNW_14_ and AgNW_28_. In Figure 5d the black arrow indicates a fibre shared by two cells and another fibre only partly enclosed by the cell (red arrow Figure 4D and E).

### Characterisation of Cell/Nanowire interaction in vitro using BSE

Backscatter electron microscopy (BSE) enabled us to visualise the interaction of THP-1 macrophage cells with AgNW. We mainly focused on the uptake/phagocytosis of the five different size categories of AgNW to identify the cut-off value, at which frustrated phagocytosis occurs *in vitro*. Figure 5A-E show differentiated THP-1 macrophages after 4 hour treatment with AgNW_3_ (A), AgNW_5_ (B), AgNW_10_ (C), AgNW_14_ (D) and AgNW_28_ (E). In Figure 5A one external AgNW_3_ can be seen (white arrow) and a few AgNW_3_ can be seen just underneath the surface of a macrophage as indicated by the black arrow (Figure 5A insert). After treatment with AgNW_5_, nanowires were fully taken up by macrophages as indicated by the white arrow (Figure 5B insert). A small proportion of cells showing incomplete uptake of AgNW_10_ fibres could be observed as indicated by the white arrow (Figure 5C) however most of the fibres were membrane bound (black/white arrow) and therefore phagocytosed by the cells. Obvious frustrated phagocytosis could be observed from a nanowire length of 14 μm (Figure 5D) and 28 μm (Figure 5E). A much greater amount of AgNW was observed protruding through cells or shared between adjacent cells indicating frustrated phagocytosis (white arrow). The number of unphagocytosed fibres per cell was quantified and significantly increased with AgNW_14_ (p > 0.05) and AgNW_28_ (p > 0.001).

### *In vivo*

#### Uptake of AgNWs in pleural macrophages from pleural lavage

**Figure 6:**
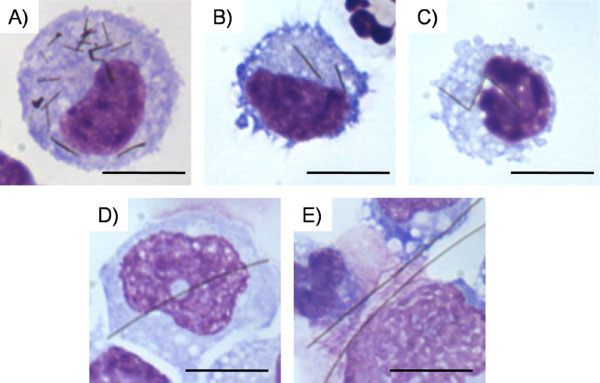
**Representative cytospin images of lavaged cells from the pleural space after 24 hour treatment with AgNWs.A**) AgNW_3_ inside a pleural macrophage showing complete phagocytosis. **B**) Pleural macrophage with a fully phagocytosed AgNW_5_ (**B**) and AgNW_10_ (**C**). **D**) A pleural macrophage with increased cell size to phagocytose AgNW_14._**E**) Frustrated phagocytosis after AgNW_28_ exposure. 3 macrophages sharing 2 long fibres. All images are shown at 100x magnification with a 10 μm scale bar.

In Figure 6A a macrophage is shown from a pleural lavage after treatment with AgNW_3_. The macrophage could readily phagocytose a number of short (3 μm) AgNWs (Figure 6A). After treatment with AgNW_5_ (Figure 6B), again the nanowires were fully taken up by the pleural macrophages in the lavage fluid. However, AgNW_10_ (C)_,_ AgNW_14_ (D) and AgNW_28_ (E) could not be fully phagocytosed by the cells anymore, leading to frustrated phagocytosis. Enlargement of the pleural macrophages (Figure 6D) could be observed. Data from the same animals as described here were utilised in Schinwald *et al*. [14] where details of the inflammatory effects accompanying these cellular changes can be obtained.

### Phagocytosis/frustrated phagocytosis *in vivo* on the parietal pleura surface 24 hour post exposure

**Figure 7:**
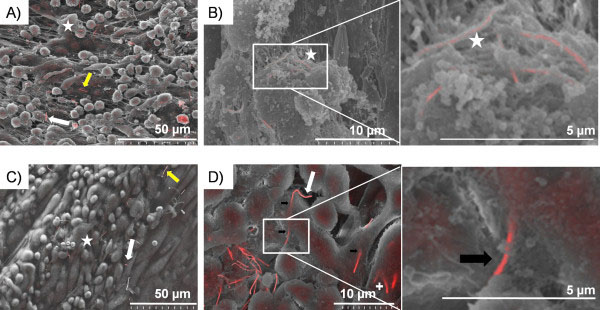
**Images of the parietal pleura surface 24 hour post pleural instillation of AgNW**_**5**_**and AgNW**_**10**_**.** Overview of the lesion area on the surface of the parietal pleura with AgNW_5_ (**A**) and AgNW_10_ (**C**). The yellow arrow indicates a nanowire which is not taken up by inflammatory cells whereas the star indicates a fibre that is fully phagocytosed but can be visualised underneath the surface of the cell. The white arrow indicates nanowires which are protruding out of cells. **B**) This figure shows AgNW_5_ that are fully phagocytosed by pleural macrophages but can be visualised underneath the surface as indicated by the star. **D**) AgNW_10_ is causing frustrated phagocytosis in a macrophage in the centre of the image. The unclosed cell membrane is indicated by the black arrows and exposed fibre surface is indicated by the white arrow. The cross on the lower right of the image indicates fibres which are largely internalised but whose tips rise vertically to penetrate the cell surface adjacent to the other long fibre.

Backscatter scanning electron microscopy examination was carried out on the macrophage accumulations on the parietal pleura samples from mice injected with AgNW_3_, AgNW_5_ and AgNW_10_ at 24 hour post intrapleural instillation in order to examine the development of interaction between the mesothelial cell layer/inflammatory cells on the surface. The dose of 5 μg per mouse used in this study lead to a significant increase in inflammation after treatment with AgNW_5_, AgNW_10_, AgNW_14_ and AgNW_28_ as previously reported [14]. A dose response was performed by increasing the dose of AgNW_3_ up to 10 μg which resulted in no significant increase in inflammation. The dose of AgNW_5_ was decreased to 1 μg and 2.5 μg whereby 2.5 μg continuously showed significant inflammation [14]. AgNW_3_ treatment was chosen to investigate if any short fibre were retained in the pleural space after intrapleural injection even though no inflammatory response was observed. AgNW_5_ and AgNW_10_ treatments were chosen since both treatments lead to a significant increase in pleural inflammation however differ in their interactions with pleural macrophages [14]. No AgNW_3_ could be observed on the parietal pleura surface 24 hour post treatments confirming that short fibres are readily cleared from the pleural space. In contrast to AgNW_3_, both AgNW_5_ and AgNW_10_ lead to an aggregation of inflammatory cells on the surface of the parietal pleura with accumulation of nanowires within the lesion area (Figure 7A,C). Most AgNW_5_ were fully phagocytosed by pleural macrophages (Figure 7A, B and insert, stars). In comparison, AgNW_10_ showed a number of fibres only partly phagocytosed and therefore leading to frustrated phagocytosis (Figure 7C,D white arrow). Some AgNW_5_ and AgNW_10_ were not taken up by macrophages as indicated by the yellow arrows (Figure 7A,C). By looking at the surface with higher magnification unclosed membrane could be visualised in pleural macrophages phagocytosing AgNW_10_ (Figure 7D and insert, black arrows).

### Phagocytosis/frustrated phagocytosis *in vivo* on the parietal pleura surface 1 week post exposure

**Figure 8:**
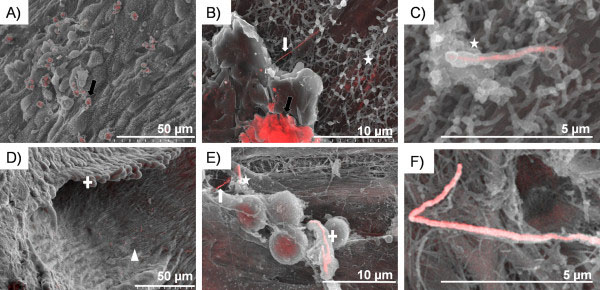
**Images of macrophages on the parietal pleura 1 week post pleural instillation of AgNW**_**5**_**and AgNW**_**10**_**.** Lesion area showing AgNW_5_ (**A**) dissolving inside macrophages, as can be seen by the loss of fibre structure (black arrow) (**B**). Intact nanofibres on the mesothelial cell layer as indicated by the white arrow (**B**) and membrane bound as indicated by the star (**B**,**C**). **D**) Lesion area appears denser and more granulomatous after 1 week treatment AgNW_10_ and occasional nanowires can be seen in these granulomatous areas as indicated by the white cross. Surprisingly, a large part of the nanowires were seen on and within the mesothelial cell layer indicated by the triangle. These nanowires appeared structurally intact whereas nanowires fully phagocytosed by macrophages as seen in **E**) indicated by the cross on the right side start to dissolve under the acidic condition within a macrophage. On the top left of this image, a fibre is partly internalised (star) and partly exposed (white arrow). **F**) AgNW_10_ which is partly membrane bound on the mesothelial cell layer.

BSEM of parietal pleura after 1 week treatment with AgNW_5_ are shown in Figure 8A,B,C and AgNW_10_ in Figure 8D,E,F. By 1 week, AgNW_5_ started to lose their integrity, presumably in the acidic conditions within a phagosome as seen in Figure 8A,B indicated by the black arrow. Non-dissolved fibres overlying the mesothelial cell layer appear intact in their morphology (Figure 8B, white arrow). Ghost-like structures of nanofibres which are covered by microvilli (Figure 8B,C star) could be seen. AgNW_10_ could be found either in accumulations of inflammatory cells which appeared denser and more granulomatous after 1 week (Figure 8D cross), or in the mesothelial cell layer (Figure 8D triangle). AgNW_10_ also started to lose integrity inside the phagocytic compartments of pleural macrophages (Figure 8E cross). Figure 8F shows AgNW_10_ partly membrane bound on the mesothelial cell layer. Data from the same animals as described here were utilised in Schinwald *et al*. [14].

### Compartmentalisation of nanowires into mesothelial cells

**Figure 9:**
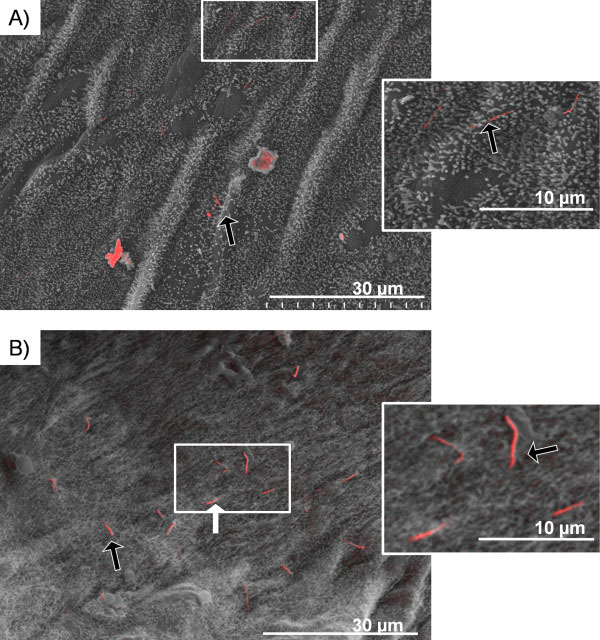
**Mesothelial surfaces of the parietal pleura 1 week post injection of AgNW**_**5**_**and AgNW**_**10**_**.** Mesothelial cell layer of the parietal pleura after AgNW_5_ (**A**) and AgNW_10_ (**B**) exposure. AgNWs can be seen on the surface of the mesothelial layer as indicated by the black arrow. These AgNW are also partly taken up by mesothelial cells as indicated by the white arrow. The morphology of the nanofibres on the mesothelial layer appears intact.

So far, we hypothesised that after a prolonged exposure to fibres in general up to 7 days, the instilled fibres in the pleural space would either be cleared from the pleural space and enter the lymphatic drainage system or be accumulated in a granulomatous lesion on the parietal pleura at stomata [15]. By using backscatter imaging, for the first time it was possible to visualise nanowires in the mesothelial cell layer of the parietal pleura. Both treatments, AgNW_5_ and AgNW_10_ (but not AgNW_3_ which were not observed as they had been cleared) showed nanofibres either on the surface of the mesothelial cells (black arrow Figure 9B) or taken up by these cells (white arrow Figure 9A,B). This is especially surprising for AgNW_5_, since pleural macrophages are able to phagocytose these fibres completely as shown in Figure 7B. By comparing the structure/shape of the nanowires associated with the mesothelial layer (Figure 9) and within macrophages (Figure 8B,E), it can quite clearly be seen that the nanowires are still intact after 1 week in the mesothelial layer whereas within the phago-lysosome of a macrophage the fibres start to degrade and lose integrity. These images show evidence that not all fibres longer than 5 μm are internalised by macrophages during the early inflammatory reaction, and that some are compartmentalised into the mesothelial layer which could lead to direct effects on the mesothelial cells and pleural diseases in a later stage if they are biopersistent.

## Discussion

The capacity of alveolar macrophages for phagocytosis and clearance of particles and fibres from the lung is of major relevance in pulmonary defence and development of lung and pleural diseases. In this study we addressed the length cut-off value at which frustrated phagocytosis occurs *in vitro* and *in vivo*. We recently defined the threshold length for inflammogenicity in the pleural space for a range of fibres including those used here [14]. However in that paper we were unable, due to restrictions on space, to fully explore the role of frustrated phagocytosis nor fully describe the disposition of fibres in the pleural space. In the present paper we were able to quantitatively compare phagocytosis/ frustrated phagocytosis of 5 different lengths of silver nanofibres ranging from 3–28 μm where inflammogenicity was determined in a previous study [14]. Thus far, the polydispersity of fibre length in samples including naturally occurring fibres and synthetic vitreous fibres prohibited the accurate investigation of the toxicity of various fibre lengths. The tight length classes of nanofibres used in this study, produced using nanotechnological methods, allowed us to perform a quantitative and qualitative comparison of the role of fibre length and frustrated phagocytosis *in vitro* and *in vivo*.

The use of a novel technique, backscatter electron microscopy allowed us to distinguish between membrane bound fibres and unphagocytosed fibres and to visualise the interaction of inflammatory cells with different length of nanofibres on the parietal pleura, the site of fibre retention *in vivo*.

The need for macrophages to internalise and therefore clear fibres from the lung and the pleural space has long been accepted [16] but the cut-off length below which complete internalisation of particles/fibres occurs is unknown. An extensive study has investigated the phagocytic capacity of murine bone marrow-derived macrophages to engulf various sizes of latex beads in a range of 13 μm to >30 μm in diameter [17]. By measuring the bead diameter they calculated a phagocytosis capacity of 19.8 μm, which is 1.44 times the actual diameter of the cell. In regard to the phagocytosis of fibres by macrophages, Ye *et al*. investigated the role of glass fibre length in TNF-α production and NF-κB activation in a mouse macrophage cell line and correlated and increase in both cytokine level and transcription factor to incomplete phagocytosis of long fibres (17 μm) whereas short fibres (7 μm) were fully phagocytosed and therefore had less expression of the measured endpoints [9]. These data correlate with our identified threshold length for frustrated phagocytosis *in vitro*, which is ≥ 14 μm.

An *in vivo* study performed by Oberdorster *et al.* assessed the clearance of small (~3 μm in diameter) and large (~10 μm in diameter) polystyrene microspheres in rat and identified minimal clearance of larger spheres after a 200 days post position period [18]. This was considered to be due to impaired clearance of particles when the macrophage particle load is 60% of its normal volume.

Frustrated phagocytosis has been implicated to play a major role in the development of an inflammatory milieu after exposure to fibres *in vitro*[19,20]. We recently demonstrated that frustrated phagocytosis is a major factor in the genesis of inflammation in the pleural space after deposition of long fibres whereby macrophages undergoing frustrated phagocytosis of the long fibres release factors that promote a potent pro-inflammatory cytokine response from adjacent mesothelial cells [20]. In addition we have demonstrated that 5 μm is the threshold for pro-inflammatory effects of fibre in the pleural space for a wide range of fibers [14]. However, so far frustrated phagocytosis has not been fully visualised *in vivo* in the pleura and the fibre length threshold for frustrated phagocytosis *in vivo* is unknown. Here we showed that the length cut off value at which long fibres can be fully phagocytosed by macrophages differs *in vitro* and *in vivo*. Whereas *in vitro* fibres of 10 μm could be completely phagocytosed, *in vivo* inflammatory cells on the parietal pleura showed incomplete uptake and frustrated phagocytosis of 10 μm fibres. There are a number of potential explanations for this anomalous difference between *in vivo* and *in vitro* length-dependent effect. The *in vivo* model is a pleural granuloma in mice and this is very different from the *in vitro* situation. Firstly the cells used *in vitro* are human cells, secondly they are a cell line and thirdly they are not in the granulomatous milieu but are spread out singly on plastic, submerged in medium with 10% serum, a very abnormal environment. We conclude that investigations on frustrated phagocytosis and its relation to the pathological effects of fibres need to be carried out *in vivo* although future work could be focussed on trying different cell lines and culture conditions with the aim of finding a better *in vitro* model that mimics the *in vivo* findings.

**Figure 10:**
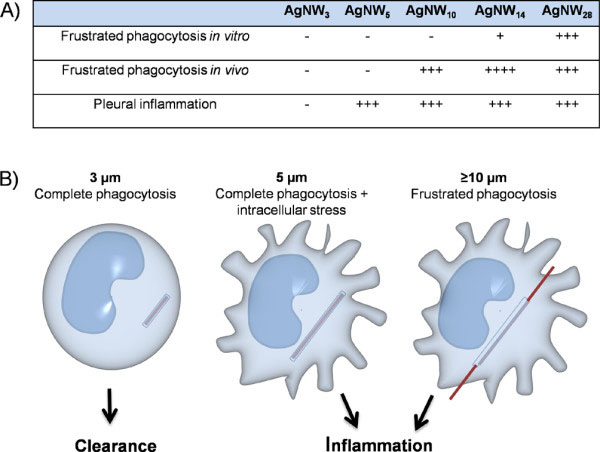
**Length categories of frustrated phagocytosis and pleural inflammation and diagrammatic representation of frustrated phagocytosis. A**) Summary of frustrated phagocytosis and pleural inflammation *in vitro* and *in vivo*. **B**) Diagrammatic representations of the length-dependent effects seen at the level of the macrophage in granulomas on the parietal pleura after instillation of fibres. These suggest that fibres 3 μm and shorter can be phagocytosed with no inflammation and cleared from the pleural space. Fibres 5 μm long can be entirely enclosed but are associated with inflammation whilst 10 μm fibres clearly cause frustrated phagocytosis and inflammation.

Interestingly, pleural injection of AgNW_5_ lead to significant inflammation in the pleural space [14] but was not associated with frustrated phagocytosis *in vivo*. These results show that frustrated phagocytosis is not solely responsible for the onset of an inflammatory reaction in the pleural space after exposure to fibrous particles and that fibres around 5 μm can be fully phagocytosed but nonetheless cause sufficient cellular stress to induce pro-inflammatory effects (Figure 10).

In this study we focused on the interaction of macrophages with nanofibres, however, our investigations of the parietal pleura revealed that intact nanofibres can also be found on the normal mesothelial cell layer after 1 week exposure. So far, we hypothesised that fibres, which are retained in the pleural space would be accumulated in lesions on the parietal pleura. In our study the mesothelial cells do not seem to be pro-inflammatory in this position as they have not attracted leukocytes to their vicinity and so their main effect may be by direct genotoxic or cytotoxic effects on the mesothelial cells. A lack of direct pro-inflammatory effect of the nanowires on the mesothelial cells would be consistent with our recent study on the interactions between long carbon nanotubes, macrophages and mesothelial cells in the genesis of inflammation. Work with long carbon nanotubes show that fibres interacting with macrophages cause the macrophages to release factors that evoke large scale production of inflammatory mediators by adjacent mesothelial cells [20]. Activated mesothelial cells produce a number of pro-inflammatory mediators including prostaglandins, nitric oxide, reactive oxygen species, cytokines and growth factors. Prolonged release of these pro-inflammatory mediators due to retention of fibres on the mesothelial surface may play a role in stimulation of tumour growth [21]. In contrast direct interaction between carbon nanotubes and mesothelial cells *in vitro* produced very little pro-inflammatory cytokine release by the mesothelial cells but did lead to significant membrane damage at higher carbon nanotube concentrations at a similar extent to that seen in macrophages [20]. A study by Adamson reported that exposure to long (≤20 μm) crocidolite asbestos fibres lead to increased pleural mesothelial cell proliferation in mice but no uptake of fibres in mesothelial cells was observed and again an indirect effect of mitogens released by cells in the sub-pleural lung tissues was implicated [22].

Therefore nanofibres that escape macrophage phagocytosis and take up residence in the mesothelium may be relatively non-inflammatory but represent a direct genotoxic threat. The interaction of AgNW with mesothelial cells changed their biopersistent characteristics, since AgNW appeared intact in their shape compared to compartmentalisation in macrophages where dissolution is observed. Silver ion measurements may not be helpful in clarifying AgNW persistence either *in vivo* or *in vitro* to the complexity of interpreting the data due to silver chloride formation as discussed in Schinwald *et al*. [14].

Our findings suggest therefore the threshold length of 5 μm is a threshold for retention of fibres that has at least 2 sequels:- 1) inflammation in the pleural space ; 2) compartmentalisation and localisation of such fibres to the mesothelial layer.

## Conclusion

The use of backscatter scanning electron microscopy enabled us to identifying frustrated phagocytosis *in vitro* and *in vivo* using metal-based nanofibre samples without the further need of nanofibre modification including radio labelling or fluorescence labelling to visualise their interaction with cells and tissue. The images and data provided in this study show a clear cut-off value for frustrated phagocytosis *in vitro* and *in vivo* (Figure 10). We could visualise the interaction of nanowires with pleural inflammatory cells *in vivo* and revealed that frustrated phagocytosis is not the only factor for the onset of pleural inflammation and 5 μm fibres can be fully enclosed but are still associated with inflammation as previously described [14]. The contrast between the *in vitro* and *in vivo* finding for the length threshold for frustrated phagocytosis suggest that THP-1 macrophages do not provide a good model for the length dependent phagocytic events that occur *in vivo* in the murine pleural space. More research may provide a better *in vitro* model that mimics the *in vivo* effects. We also visualised the interaction of mesothelial cells with nanofibres and described a novel compartmentalisation of them in mesothelial cells which appeared to show different biopersistence characteristics compared to compartmentalisation in macrophages. However this needs to be confirmed by more quantitative studies rather than the merely descriptive data that are presented here.

## Materials and methods

### Backscatter electron signals by scanning electron microscopy

Elements with high atomic number (*Z*) such as silver reflect or back-scatter electrons more strongly than the lower *Z* light elements (predominantly H, C, N, O, P) of which cells are composed. Back-scattered electron imaging (BSE) is therefore a useful way to study frustrated phagocytosis since it provides high-contrast detection of nanowires, allowing clear discrimination between the nanofibres and other cellular features. Since the BSE signal from the fibres is attenuated by overlying cellular material, the method allows a clear distinction to be made between fibres that are, or are not, membrane-bounded, and can allow detection of fully phagocytosed fibres, which would not otherwise be detected in an SE image, provided they are at shallow depth within the cell.

This principle has been used widely for detection of colloidal gold markers in immunocytochemistry [23,24].

### Fibre panel and size distribution

The fibre panel consisted of five distinct length classes of silver nanowires, hereafter referred to as AgNW_3_, AgNW_5_, AgNW_10_, AgNW_14_ and AgNW_28_ whereby the subscript numbers indicate the average length of the nanowires. AgNW length diameter, contamination, soluble metal content, endotoxin level and dispertion was characterised as described previously by Schinwald *et al.*[14] (Figure 2). The samples were kindly provided by Seashell Technology, San Diego and synthesised using a polyol process as decribed in the US patent number 7,922,787 B2.

For light microscopy images 1 mg/ml of AgNWs were dispersed in 0.5% bovine serum albumin (BSA; Sigma-Aldrich, Poole, UK) and 10 μl of suspension was mixed in equal volume of glycerol (Sigma-Aldrich, Poole, UK) to reduce the flow of AgNW. The suspension was placed on a glass slide, covered with coverslip and images taken using QCapture Pro software (Media Cybernetics).

### *In vitro* study

#### *In vitro* macrophages exposed to fibres of different lengths

##### Cell culture

The immortalised human monocytic cell line THP1 was used for *in vitro* studies. Cells were cultured in RPMI media supplemented with 10% heat inactivated FBS, 1% penicillin/streptomycin and 1% L-Glutamine (PAA, Austria). Prior to each treatment the cells were seeded in 24-well plates at a density of 0.5*10^6^/ml in 500 μl medium containing 10% FBS and 10 ng/ml phorbol 12-myristate 13-acetate (PMA) (Sigma) for 2 days at 37°C in 5% CO_2_ atmosphere [25]. Fibres were uniformly dispersed in cell culture medium (RPMI 1640) supplemented with 1% penicillin/streptomycin and 1% L-Glutamine (PAA, Austria) and 0.5% bovine serum albumin (BSA; Sigma-Aldrich, Poole, UK) and briefly vortexed. Cells were treated with AgNW equalised to fibre number since fibre exposure is regulated on the basis of the fibre number and so relative potency needs to be determined on a per-fibre basis. To equalise for fibre number a dose of 2 μg/cm^2^ for AgNW_14_ was chosen as the standard in vitro dose based on previous measurement of membrane integrity and proliferation. Based on 2 μg/cm^2^ for AgNW_14_, concentrations for the other length classes AgNW panel were calculated assuming that fibres thickness was constant in the different length classes (Table 1).

### Measurement of membrane integrity and proliferation of THP-1

Cells were seeded at a concentration of 0.5*10^6^ cells/ml and treated for 24 hours as described above. TritonX (Sigma) was used as a positive control for cell death and was added at a final concentration of 0.1% for 30 mins. After the treatment supernatant was centrifuged for 5 mins at 2000 rpm, transferred and centrifuged again for 5 mins at 13000 rpm**.** The conversion of lactate to pyruvate was detected using the Cytotoxicity Detection Lactate Dehydrogenase kit (Roche Diagnostics Ltd., Burgess Hill, UK) following the manufacturer’s instructions. A microplate reader (BioTek® SynergyHT) was used to measure the optical density at 490 nm. Results are given as the mean ± SEM of 5 independent experiments.

Cells in the culture dish were used to measure their proliferation and metabolic activity via a chemical reduction of AlamarBlue® (Invitrogen). 150 μl of PBS and 15 μl of AlamarBlue® was added to each well and incubated for 3 hours at 37°C in 5% CO_2_ atmosphere. Absorbance was monitored at 570 nm and 600 nm as a reference wavelength. Data are normalized to 600 nm value. Results are given as the mean ± SEM of 5 independent experiments.

### Preparation for BSE

THP-1 cells were differentiated as described above and seeded into 24 well plates on Thermanox® Plastic Coverslips (NUNC™, Rochester, NY USA) at a density of 0.5*10^6^/ml. The cells were treated for 4 hours using concentration as described above at 37°C in 5% CO_2_ atmosphere. After the treatment they were washed 5× with 0.1 M sodium cacodylate (pH 7.2) buffer. Overnight fixation was done in 3% glutaraldehyde/ 0.1 M sodium cacodylate (pH 7.2) buffer. After fixation the cells adherent to the coverslips were washed three times in sodium cacodylate buffer.

### Bright field microscopy

THP-1 cells were differentiated as described above in a μ-dish (35 mm) (ibidi, Germany) and treated for 4 hours as described above. Brightfield microscope images were taken using Leica confocal laser scanning microscope SP5 at a 60× oil immersion objective lens.

### *In vivo* study

#### Intra pleural injection of fibres

Fibres were uniformly dispersed in 0.5% bovine serum albumin (BSA; Sigma-Aldrich, Poole, UK)/saline at a concentration of 50 μg/ml which equates a dose of 5 μg per mouse and injected into the pleural cavity of female C57BI/6 mice (aged 8 weeks) at a volume of 100 μl per mouse as described previously by Schinwald *et al*. [14]. We used a sleeve close to the tip of a 27 G needle to prevent it penetrating beyond the pleural space into the lung [13]. Mice were euthanized after 24 hours (n = 4) and 7 days (n = 4) by asphyxiation in 100% CO_2_.

#### Lavage of pleural space

The pleural space was lavaged with three 1 ml washes of sterile saline and kept on ice. To separate the cellular fraction from the supernatant the lavage fluid was centrifuged for 5 minutes at 2000 rpm at 4°C in a Mistral 3000i centrifuge (Thermo Fisher Scientific, Inc., MA, USA). Cyto-centrifugation with following Diff-Quik staining using Diff-Quik stainset (Dade Behring Gmbh, Marburg, Germany) were prepared for visualising uptake of fibres in pleural macrophages.

### Preparation parietal pleura for BSE

#### Tissue dissection

The lower right posterior portion of the chest wall, approximately an area of 1 cm × 0.5 cm along the spine was cut out from the mice after lavage, washed in ice-cold saline and fixed for 4 hours in 30% formalin. The tissue was excised from the surrounding tissue and fixed with 3% glutaraldehyde in 0.1 M Sodium Cacodylate buffer (pH 7.3) for 3 hours then washed in three 10 minute changes of 0.1 M Sodium Cacodylate buffer.

### Cell and tissue preparation for BSE

Fixed samples were dehydrated in 50%, 70%, 90% and 100% normal grade acetones for 10 minutes each, then for a further two 10-minute changes in analar acetone. Dehydrated samples were critical point dried and mounted on SEM aliminium stubs and rotary-coated with about 8 nm of carbon in an Edwards 306A vacuum coating system (Edwards High Vacuum, Crawley, UK).

### BSE

SEM of carbon-coated specimens was carried out using a Hitachi 4700 II field emission SEM (Hitachi High-Tech, Maidenhead, UK) at a beam accelerating voltage of 10 kV and a working distance of about 8 mm. Secondary electron (SE) and BSE images were taken simultaneously using an annular YAG crystal BSE detector and the upper SE detector to produce perfectly-synchronised image pairs. The two images were superimposed using Adobe Photoshop. The SE and BSE image were converted to grayscale, the BSE image was pasted into the SE image by using the layer function “lighten”. This newly merged image and the SE image were converted to RGB mode, and overlayed by pasting the red channel of the BSE image into the red channel of the greyscale SE image, thus colour coding in red the strong BSE signal from the nanowires, the SE image appearing in grey.

### Methodology for quantifying unphagocytosed fibres

Image-Pro plus software (Media Cybernetics Inc., MD, USA) was used to measure the amount AgNW outside macrophages. The intensity of red pixels of the nanofibres differs if the fibre is taken up by a cell and therefore covered by a cell layer (lower intensity) or if the fibres is unphagocytosed (higher intensity). Using Image-Pro software, it was possible to specifically select the red pixel intensity of unphagocytosed cells which was expressed as object and area count. The number of objects (unphagocytosed fibres) was divided by the number of cells per image and expressed as unphagocytosed fibres per cell. Approximately 100 cells per image were counted (n = 3).

### Statistical analysis

All data are shown as the mean ± s.e.m. and these were analysed using one-way analysis of variance (ANOVA). Multiple comparison were analysed using Tukey-HSD method and in all cases (GraphPad InStat Software Inc., CA, USA).

## Abbreviations

AgNW: Silver Nanowires; NiNW: Nickel nanowires; BSM: Backscatter scanning electron microscopy; LDH: Lactate dehydrogenase.

## Competing interests

The authors declare that they have no competing interests.

## Authors’ contributions

A.S. conceived and designed the experiments, analysed the data and wrote the manuscript. K.D. initiated the study, oversaw all experimental work and contributed to manuscript preparation. All authors read and approved the final manuscript.
